# Preventing Mitochondrial Fission Impairs Mitochondrial Function and Leads to Loss of Mitochondrial DNA

**DOI:** 10.1371/journal.pone.0003257

**Published:** 2008-09-22

**Authors:** Philippe A. Parone, Sandrine Da Cruz, Daniel Tondera, Yves Mattenberger, Dominic I. James, Pierre Maechler, François Barja, Jean-Claude Martinou

**Affiliations:** 1 Department of Cell Biology, University of Geneva, Geneva, Switzerland; 2 Department of Cell Physiology and Metabolism, Geneva University Medical Center, Geneva, Switzerland; 3 Laboratoire de Bioenergetique et Microbiologie, Universite de Geneve, Geneve, Switzerland; Baylor College of Medicine, United States of America

## Abstract

Mitochondria form a highly dynamic tubular network, the morphology of which is regulated by frequent fission and fusion events. However, the role of mitochondrial fission in homeostasis of the organelle is still unknown. Here we report that preventing mitochondrial fission, by down-regulating expression of Drp1 in mammalian cells leads to a loss of mitochondrial DNA and a decrease of mitochondrial respiration coupled to an increase in the levels of cellular reactive oxygen species (ROS). At the cellular level, mitochondrial dysfunction resulting from the lack of fission leads to a drop in the levels of cellular ATP, an inhibition of cell proliferation and an increase in autophagy. In conclusion, we propose that mitochondrial fission is required for preservation of mitochondrial function and thereby for maintenance of cellular homeostasis.

## Introduction

Mitochondria form a highly dynamic tubular network in eukaryotic cells. The organisation, shape and size of these organelles is regulated by movements along the cytoskeleton but also by frequent fission and fusion events [1], [2]. Evolutionary conserved cellular components that regulate mitochondrial fission and fusion have been identified in yeast, fly and mammals [3]. Mitochondrial fission relies on a large dynamin related GTPase called Drp1 (Dnm1p in yeast). Drp1 is located mostly in the cytosol of mammalian cells and a pool of the protein translocates to the mitochondrial tubules where it assembles, through its interaction with hFis1 [4], [5], into foci at future fission sites [6], [7]. Inhibition of Drp1 function using either expression of DrpK38A, a dominant negative mutant defective in GTP binding, or RNA interference, leads to the formation of a highly fused and tubular mitochondrial network, thus implicating Drp1 in mitochondrial fission [6], [8]. Mitochondrial fusion in mammalian cells depends on a distinct set of evolutionary conserved components, namely the dynamin-related GTPases Mfn1,2 and OPA1 (for reviews see [3]).

Mitochondrial dynamics is clearly important in cellular homeostasis. Mutations in the *OPA1* or *MFN2* genes respectively cause the most commonly inherited optic and peripheral neuropathies (autosomal dominant optic atrophy and Charcot-Marie-Tooth disease; [9], [10]). Studies on cultured mammalian cells have shown that formation of a reticular mitochondrial network is important for proper mitochondrial calcium buffering and for propagating intra-mitochondrial Ca^2+^ waves [11], [12]. Mitochondrial fusion is required for the maintenance of mitochondrial DNA (mtDNA; [13]) and inhibiting this process has been shown to reduce the activity of the electron transfer chain (ETC; [14]) and to reduce mitochondrial metabolism [15]. The role of mitochondrial fission, on the other hand, is less clear. It has been proposed to be required for apoptosis [16], [17], although this proposal has recently been challenged [18]–[20].

In this study, we set out to determine the role of mitochondrial fission in mitochondrial and cellular homeostasis. Here, we show that preventing mitochondrial fission by down-regulating expression of Drp1 leads to mitochondrial dysfunction, an increase in cellular reactive oxygen species (ROS) and a loss of mtDNA which correlates with a depletion of cellular ATP, inhibition of cell proliferation and autophagy.

## Results

### Depletion of Drp1 in HeLa cells leads to mitochondrial dysfunction

In order to investigate the role of mitochondrial fission in mitochondrial and cellular homeostasis, RNA interference was used to down-regulate expression of Drp1. To this end, a small hairpin RNA (shRNA) targeting the Drp1 transcript was synthesised *in vivo* by means of the shRNA expression vector pRETRO-SUPER (D1; [21]). As a control, a similar construct expressing a shRNA targeting the luciferase transcript was used (Ctrl). As shown in Fig. S1A, protein levels of Drp1 were strongly reduced at 96 h after transfection of HeLa cells with the D1 construct. At the same time point, analysis of mitochondrial morphology by immunofluorescence using an anti-TOM20 antibody, revealed highly fused and interconnected mitochondria (Fig. S1B), confirming that Drp1 is required for mitochondrial fission [7].

To assess whether mitochondrial fission is required for the maintenance of mitochondrial homeostasis, mitochondrial functional parameters were measured in Drp1-depleted cells using flow cytometry. Mitochondrial inner membrane potential (ΔΨm) is a critical aspect of mitochondrial homeostasis. We therefore determined if ΔΨm was affected in Drp1-depleted cells by quantifying fluorescence of the cationic dye JC-1 by flow cytometry.

**Figure 1:**
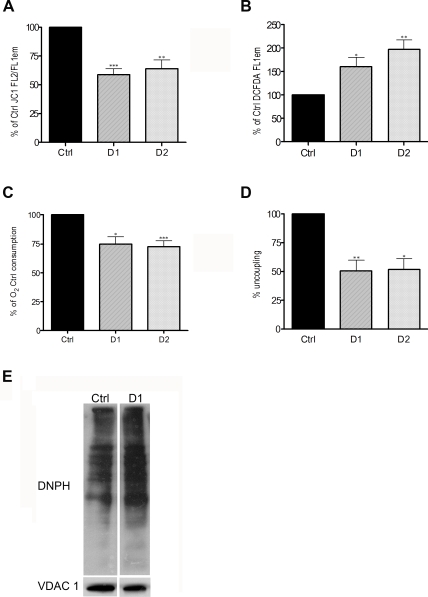
Depleting HeLa cells of Drp1 leads to mitochondrial dysfuntion. A. HeLa cells were transiently transfected with the Ctrl, D1 or D2 constructs, selected with puromycin for 24 h, collected 96 h after transfection and stained with JC1 for flow cytometric analysis. The results are expressed as a percentage of the ratio between red and green emissions (FL2/FL1) in Ctrl cells and represent the mean+SEM from 7 independent experiments (***: P<0.0005). B. HeLa cells treated as in A. were stained with DCFDA for flow cytometric analysis. The results are expressed as a percentage of the DCFDA green fluorescence (FL1) in Ctrl cells and represent the mean+SEM from 7 independent experiments (*: P<0.05). C. HeLa cells were infected with Ctrl, D1 or D2 retroviruses, selected with puromycin for 24 h, and collected 144 h after the infection. Oxygen consumption was expressed as a percentage of the O_2_ consumption in Ctrl cells. The results represent the mean+SEM from 4 independent experiments (*: P<0.05). D. Oxygen consumption was assessed in mitochondria isolated from HeLa infected with the Ctrl, D1 or D2 retroviruses. The percentage uncoupling was determined by dividing the amount of oxygen consumption in state IV (in the presence of Succinate only) by that in state III respiration (in the presence of Succinate and ADP). The results represent the mean+SEM from 3 independent experiments (**: P<0.005). E. DNP-derivatized protein lysates of mitochondria isolated from Hela cells treated as in A were separated by polyacrylamide gel electrophoresis followed by Western blotting analysis using the indicated antibodies.

JC-1 indicates mitochondrial polarization by shifting its fluorescence from green (FL1; ∼525 nm) to red (FL2; ∼590 nm) in a potential-sensitive manner due to concentration-dependent formation of red fluorescent J-aggregates. As shown in Fig. 1A, ΔΨm (expressed as the ratio of FL2/FL1 in order to account for variations in mitochondrial volume) is significantly lower in Drp1-depleted cells, (58,8%±SEM 5.2, compared to Ctrl cells). The same results were obtained when a different potentiometric dye, namely TMRE, was used to determine ΔΨm (data not shown). We next investigated if the production of ROS was altered upon inhibition of mitochondrial fission. The levels of ROS were measured by flow cytometry in Drp1-depleted cells using carboxy-H_2_DCFDA which upon exposure to oxidative species is oxidized to the green fluorescent probe carboxy-DCF. As shown in Fig. 1B, Drp1-depleted cells loaded with carboxy-H_2_DCFDA emitted significantly more in the green (FL1) spectra (160.5%±SEM 19.8, compared to Ctrl cells). The ΔΨm and ROS levels were similarly decreased and elevated respectively when Drp1 was depleted in HeLa cells using an shRNA, D2, that targets a different region of the Drp1 transcript (Fig. S1A, B and Fig. 1A, B).

Together these results suggest that the function of mitochondria in Drp1 depleted cells may be impaired and that oxidative damage in these organelles may be increased. We therefore examined the principal activity of the organelle, namely mitochondrial respiration, in D1 and D2 transfected cells using standard Clark electrode oxymetry. As shown in Fig. 1C, oxygen consumption in growth media (at pH 7.5) was significantly decreased in intact live Drp1-depleted cells (74.7%±SEM 6.1 and 72.7%±SEM 4.9 for D1 and D2 respectively compared to Ctrl cells). Furthermore, when respiration was assessed in isolated organelles, mitochondria from Drp1-depleted cells not only consumed less oxygen in the presence of succinate and ADP (state III; data not shown) but were also markedly uncoupled, as judged by the increased ratio of State IV (succinate without ADP) to State III (Fig. 1D). These results suggest that depleting cells of Drp1 causes mitochondrial dysfunction. To determine if this correlates with mitochondrial oxidative damage, we determined the level of protein carbonyls in mitochondria isolated from D1 and Ctrl cells through a reaction with 2,4-dinitrophenylhydrazine (DNPH). As shown in Fig. 1E, the level of protein oxidation in D1 mitochondria was 37% higher compared to Ctrl.

### Preventing mitochondrial fission in HeLa cells leads to a decrease in cellular ATP content, inhibition of cell proliferation and autophagy

**Figure 2:**
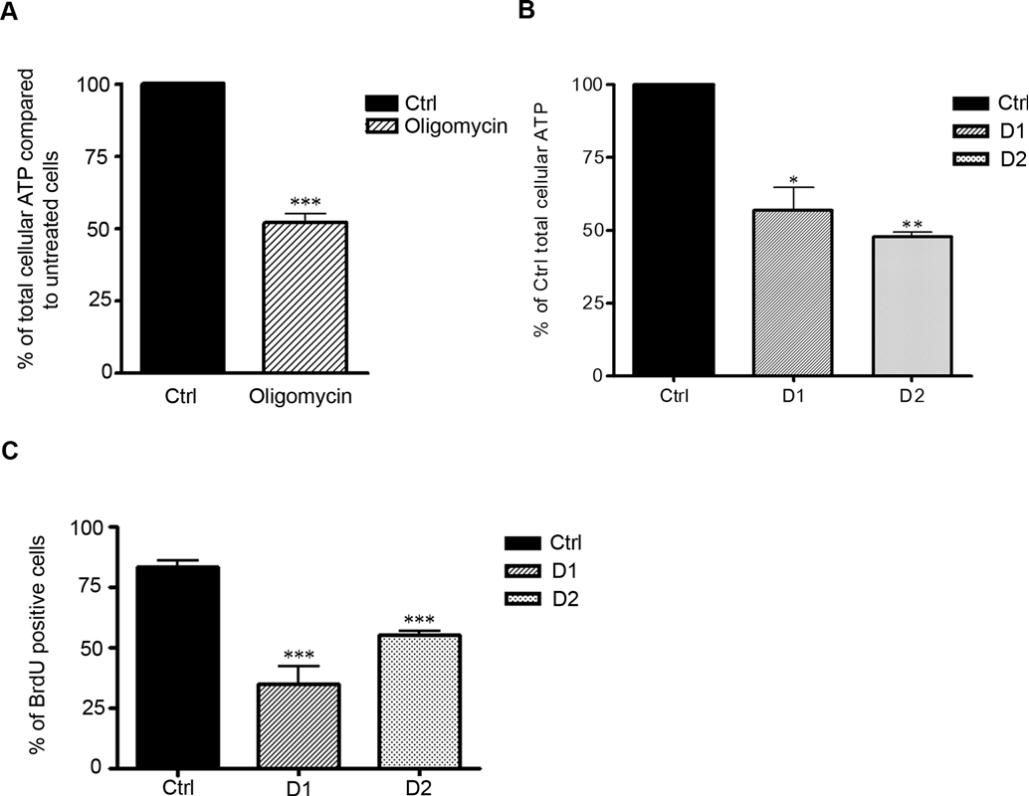
Inhibiting mitochondrial fission in HeLa cells leads to drop in ATP levels and a proliferative arrest. A. HeLa cells were treated with the F_1_F_0_ ATP synthase inhibitor oligomycin (10 µM) and total cellular ATP levels were determined after 2 h of treatment. ATP levels after oligomycin treatment are expressed as the percentage of the ATP level in untreated HeLa cells. Results are calculated from 3 independent experiments+SEM (***: P<0.0005). B. HeLa cells were transiently transfected with the Ctrl, D1 or D2 constructs, selected with puromycin for 24 h, collected 96 h to measure total cellular ATP content. The quantity of ATP in D1 and D2 cells was expressed as a percentage of the ATP in Ctrl cells. The results represent the mean+SEM from 3 independent experiments (*: P<0.05). C. 96 h after transfection, Hela cells treated as in B. were grown in the presence of 10 µM BrdU for a further 18 h, fixed and stained with a BrdU antibody. The percentage of cells positively stained with BrdU was quantified from 3 independent experiments+SEM (***: P<0.0005).

Mitochondria are a major source of ATP in all cell types including HeLa cells, as inhibiting mitochondrial ATP production in these cells (using the ATP synthase inhibitor oligomycin) leads to a 50% decrease in total cellular ATP levels (Fig. 2A). Since mitochondrial respiration is significantly impaired in Drp1-depleted cells, we examined the levels of ATP in HeLa cells transfected with Drp1 RNAi. As shown in Fig. 2B, in D1 and D2 cells, ATP levels were reduced by approximately half (44.4%±SEM 7.7 and 53.7%±SEM 2.0 for D1 and D2 respectively) compared to Ctrl cells. Therefore, the mitochondrial dysfunction observed in cells depleted of Drp1 leads to a significant drop in total cellular ATP levels. Importantly, we have previously reported that depleting HeLa cells of Drp1 using the D1 or D2 constructs does not induce cell death (at the time points tested) when assessed by flow cytometric analysis of Annexin V and propidium iodide stained cells [18]. Therefore, since the drop in total cellular ATP at 96 hrs after transfection of HeLa cells with the D1 or D2 constructs was compatible with cell survival, we determined if it affected cell propagation. We investigated the rate of proliferation of Drp1-depleted cells by quantifying the uptake of the thymidine analog bromodeoxyuridine (BrdU), which is incorporated into newly synthesized DNA strands of actively cycling cells. As shown in Fig. 2C, the number of cells that incorporated BrdU was significantly decreased in D1 and D2 transfected cells compared to Ctrl (35.0%±SEM 7.0 for D1, 55.4%±SEM 1.0 for D2 and 83.4%±SEM 2.4 for Ctrl).

**Figure 3:**
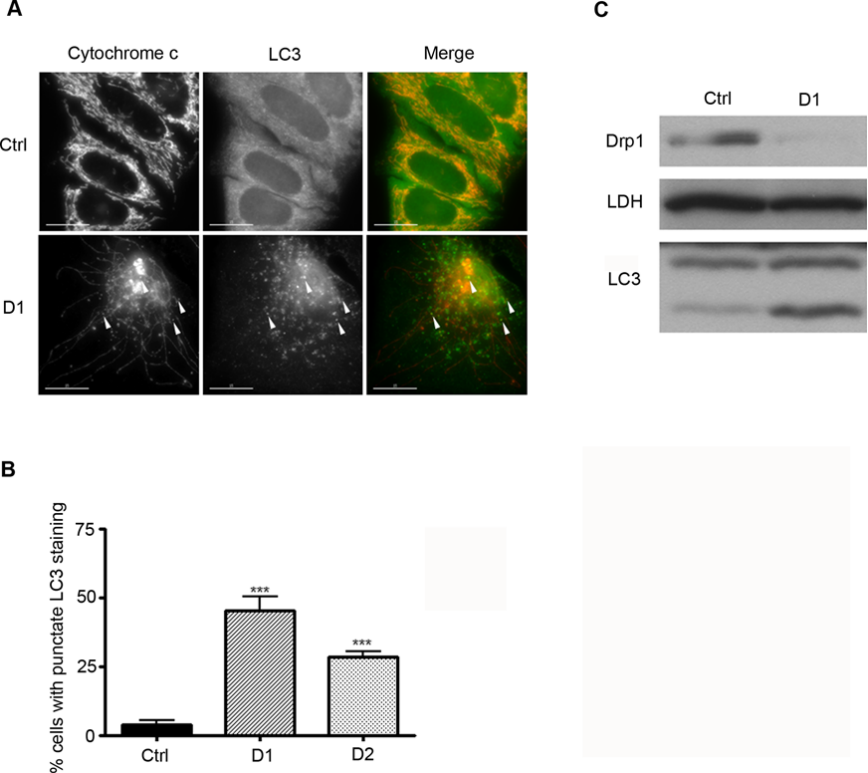
Inhibiting mitochondrial fission in HeLa cells triggers autophagy. A. HeLa cells were transiently transfected with the Ctrl, D1 or D2 constructs, selected with puromycin for 24 h, fixed 96 h post-transfection and co-stained with antibodies against LC3 and cytochrome c. The scale bar corresponds to 15 µm. B. The number of cells in A. with a punctate LC3 pattern was quantified and the results represent the mean+SEM from 3 independent experiments (***: P<0.0005). C. Cells treated as in A. were collected 96 h after transfection for Western blotting analysis using the indicated antibodies.

Autophagy is typically activated by fasting and nutrient deprivation [22]. Since preventing mitochondrial fission in HeLa cells led to depletion of ATP, we assessed if this was accompanied by an autophagic response in Drp1-depleted cells. Microtubule-associated protein light chain 3 (LC3) is a widely used marker to monitor autophagy. Upon the induction of autophagy LC3 relocalises to the newly formed autophagosomes, changing from a diffuse to a punctate pattern as observed by immunostaining, and is modified to a more rapidly migrating form that can be observed on SDS-PAGE [23]. As shown in Fig. 3A, the immunostaining pattern of D1 transfected cells with an anti-LC3 antibody was distinctively punctate compared to the diffuse staining pattern observed in Ctrl cells (45.2%±SEM 5.1 and 3.8%±SEM 1.7 of the D1 and Ctrl cells respectively had a punctate LC3 immunostaining pattern, Fig. 3B). Similar results were obtained in D2 cells (Fig. 3B). Furthermore, by immunoblotting the protein levels of the more rapidly migrating form of LC3 (induced upon upregulation of autophagy) were significantly increased in total lysates of Drp1-depleted cells compared to Ctrl cells (Fig. 3C). These results show that inhibition of mitochondrial fission in HeLa cells induces autophagy. Of note, from the evidence presented in Fig. 3A very few of the LC3 positive vesicles colocalise with the mitochondrial marker cytochrome c (arrowheads on merged Fig. 3A) and LC3 positive punctae colocalise only with small round mitochondria.

### Preventing mitochondrial fission in HeLa cells leads to loss of mitochondrial DNA

**Figure 4:**
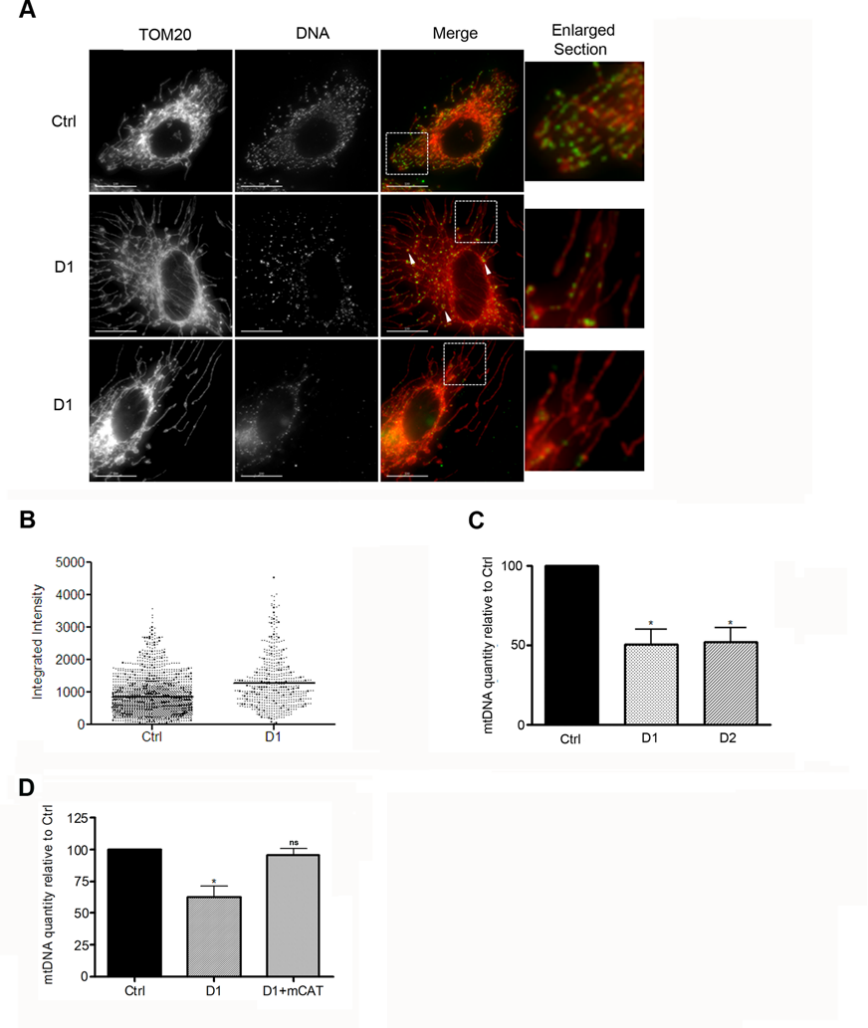
Inhibiting mitochondrial fission in HeLa cells leads to a loss of mtDNA. A. HeLa cells were transiently transfected with the Ctrl, D1 or D2 constructs, selected with puromycin for 24 h, fixed 96 h post-transfection and stained with antibodies against DNA and TOM20. The scale bar corresponds to 15 µm. B. The integrated intensity of individual mtDNA nucleoids of the cells in A. was quantified from maximal projection images from three independent experiments and the results represent the intensity of 1824 nucleoids from 10 Ctrl cells and 584 nucleoids from 9 D1 cells. C. Cells treated as in A. were collected 96 h after transfection and DNA was extracted for quantitative PCR amplification of the 12S ribosomal RNA small subunit mitochondrial gene. The results are expressed as a percentage of the mtDNA in Ctrl cells and represent the mean+SEM from 3 independent experiments (*: P<0.05). D. HeLa cells transfected with the indicated constructs were treated as in A. and processed as in C. The results are expressed as a percentage of the mtDNA in Ctrl cells and represent the mean+SEM from two experiments (*: P<0.05, ns: not significant).

It is now accepted that excessive mitochondrial fission, induced by a loss of fusion protein such as mitofusins or Fzo, leads to a loss of mtDNA in yeast and in mammalian cells [24], [25]. Therefore we set out to determine if inducing excessive fusion of the mitochondrial network by depleting HeLa cells of Drp1 led to alterations in the levels of mtDNA. Mitochondrial DNA nucleoids were stained with an anti-DNA antibody in D1 and Ctrl cells. As shown in Fig. 4A the DNA-specific antibodies labeled punctate structures that have been previously reported to correspond to mtDNA nucleoids [26]. These punctae are distributed throughout the entire mitochondrial network in Ctrl cells, as shown by co-staining with the outer-mitchondrial membrane marker TOM20. However, in Drp1-depleted cells most of the long tubular mitochondria were devoid of DNA punctae and several intensely stained mtDNA nucleoids were often clustered in discreet regions of the mitochondrial tubule or in large vesicular mitochondria close to the nucleus (see arrowheads in Fig. 4A). This later observation was confirmed when the average fluorescent intensity of individual mtDNA nucleoids was quantified and found to be 1.5 fold higher in D1 cells (Fig. 4B). These data show that in Drp1-depleted cells extensive portions of the mitochondrial network are devoid of mtDNA, or contain quantities of mtDNA that cannot be detected by immunostaining, and that the remaining mtDNA nucleoids often cluster together and contain more copies of the mtDNA molecules. In order to determine quantitatively whether there was a change in the total levels of mtDNA in Drp1-depleted cells, we used quantitative PCR amplification of the 12S ribosomal RNA small subunit mitochondrial gene as previously reported [26]. As shown in Fig. 4C, the levels of 12S rRNA gene were significantly lower in D1 cells (50.6%±SEM 9.1) compared to Ctrl cells. Depleting Drp1 from HeLa cells using the D2 shRNA also led to a similar decrease in mtDNA (by 51.9%±SEM 8.8). Altogether, these results suggest that inhibiting mitochondrial fission in HeLa cells leads to a loss of mtDNA. In Fig. 1 we provide evidence that, in HeLa cells depleted of Drp1, ROS levels and mitochondrial protein oxidation are significantly increased. In order to determine if oxidative damage is a cause of mtDNA loss in D1 cells we tested whether expression of a mitochondrially targeted form of catalase (mCAT) could reverse mtDNA depletion in D1 cells. Previous studies have shown that mitochondria isolated from mCAT transgenic animals produce less H_2_O_2_ and have lower levels of mtDNA oxidative damage [27]. As shown in Fig. 4D, the levels of 12S rRNA gene in HeLa cells cotransfected with D1 and mCAT were not significantly different from those in Ctrl cells. Altogether, these results suggest that inhibiting mitochondrial fission in HeLa cells leads to a loss of mtDNA and that this depletion can be prevented by the expression of an H_2_O_2_ scavenger at the mitochondria.

## Discussion

In this study we report that depleting cells of Drp1 leads to mitochondrial dysfunction, an increase in cellular ROS levels and loss of mtDNA which is accompanied by a drop in cellular ATP levels, a proliferative arrest and autophagy.

Recent studies have reported that mammalian cells depleted of Drp1 produced less ATP and consumed less oxygen [20], [28], thus supporting some of our conclusions. In addition we find that there is a significant increase in ROS levels, that mitochondrial respiration is markedly uncoupled and that mtDNA is lost when mitochondrial fission is inhibited by depletion of Drp1. This raises the question as to what is the primary cause of these mitochondrial alterations and in light of the results presented here we propose that Drp1 dependent fission protects the mitochondria from excessive damage. It is widely accepted that the vast majority of cellular ROS can be traced back to the mitochondria, therefore making this organelle a primary target for oxidative damage which has been shown to result in mitochondrial dysfunction and depletion of mtDNA [29]–[33]. Interestingly, it has been reported that inducing a localized increase in ROS levels along mitochondrial tubules results in a drop in ΔΨm in the targeted zone which is followed by the fragmentation of this portion of the mitochondrial network [34], [35]. In this study we show that preventing mitochondrial fragmentation leads to mitochondrial oxidative damage and mtDNA loss and that expressing a H_2_O_2_ scavenger at the mitochondria in D1 cells prevents the loss of mtDNA. Therefore, altogether these results suggest that mitochondrial fission is required to isolate (through fragmentation) damaged regions of the mitochondrial tubule. This would prevent accumulation of damage in the mitochondrial network and preserve the function, as well as the genome, of the organelle.

Alternatively, the primary cause of the mitochondrial alterations observed in Drp1 deficient cells could be the loss of mtDNA, which would lead to loss of respiratory capacity, a drop in membrane potential and an increased production of ROS. It is possible that depleting cells of Drp1 may directly or indirectly affect mtDNA replication, distribution or segregation. In support of this hypothesis we find that mtDNA, in addition to being depleted, accumulates in discreet sections of the mitochondrial tubules and that mtDNA nucleoids on average contain more mtDNA molecules in D1 cells compared to Ctrl (Figure 4). However, it is unlikely that Drp1 is directly involved in aspects of mtDNA maintenance since it has been shown not to colocalise with mtDNA nucleoids in HeLa cells [36]. Furthermore, the levels of TFAM and mtSSB, proteins involved in mtDNA maintenance, were unchanged in D1 compared to Ctrl cells (data not shown). Nevertheless, we cannot exclude that depleting cells of Drp1 may affect the function or level of other proteins involved in mtDNA maintenance, such as the DNA polymerase PLOG, or indirectly alter mtDNA distribution by preventing fission of the mitochondrial tubule.

In this study we show that the mitochondrial dysfunction resulting from the inhibition of mitochondrial fission leads to cell proliferation arrest and autophagy. Both these events are likely to be due to the drop in ATP levels in Drp1-depleted cells. Several studies have shown that inhibiting mitochondrial function, either by decreasing mitochondrial protein synthesis or by inhibiting respiration, led to ATP depletion and growth arrest [37]–[39]. Furthermore, we have found that treating HeLa cells with oligomycin or the mitochondrial uncoupler CCCP leads to an arrest in the cell cycle (data not shown).

Autophagy is widely associated with conditions of nutrient starvation [22] and thus the increase in autophagy in Drp1-depleted cells possibly occurs in an effort to refuel the cellular ATP production. Although autophagy is known to remove damaged mitochondria [22], [40], we find that autophagy occurring in D1 cells does not primarily target mitochondria (Fig. 3A). The only instance in which there is co-localisation between the autophagic and mitochondrial markers in Drp1-depleted cells is when the mitochondria are small and round (arrowheads Fig. 3A). This is in agreement with results showing that Drp1-overexpression (resulting in fragmentation of the mitochondrial network) promoted disappearance of mitochondria during apoptosis, while overexpression of dominant-negative Drp1^K38A^ prevented this elimination of the organelle [41], [42]. Therefore, although most of the long tubular mitochondria in Drp1-depleted cells are likely to be damaged, these organelles are too large to be eliminated by mitophagy and it is only when small sections of the mitochondrial tubules separate that they can be targeted for autophagic degradation.

In conclusion, we have shown that an active mitochondrial fission machinery is required for maintenance of mitochondrial function and for preservation of its genome.

## Materials and Methods

### Materials

Most chemical compounds were purchased from Sigma-Aldrich. JC1, TMRE and DCFDA were from Molecular Probes (Invitrogen). The following antibodies were used: LDH-A (Sigma), LC3 (MBL), GAPDH (6C5, Abcam), Drp1 (DLP1, Transduction Laboratories), BrdU and cytochrome *c* (BD Pharmingen), DNPH and DNA (Chemicon), VDAC1 (Calbiochem), TOM20 (Santa-Cruz).

### Cell Culture and transfections

HeLa CCL-2 cells (purchased from the European Collection of Cell Cultures) and 293T cells were cultured in high-glucose Dulbecco's minimal essential medium with 10% fetal bovine serum, 100 U/ml penicillin, 0.1 mg/ml streptomycin, and 2 mM glutamine and maintained in 5% CO_2_ at 37°C. For transient transfections cells were plated in culture dishes 45 min before transfection and transfected using a calcium phosphate coprecipitation method [43]. At 24 h after transfection the cells were washed once with Tris-buffered saline (TBS) and grown in fresh medium supplemented with 3 µg/ml puromycin (Calbiochem) for 24 h to select for the transfected cells. The cultures were then washed with phosphate-buffered saline (PBS) and incubated in fresh growth medium until the start of the experiment.

The mitochondrial catalase construct was kindly provided by Dr. Peter Rabinovitch (University of Washington, Seattle, USA).

### RNA interference and Retroviruses

Down-regulation of Drp1 in HeLa was achieved by RNA interference using a vector-based shRNA approach [21]. The target sequences were 5′-GCAGAAGAATGGGGTAAAT-3′ for D1 (nucleotides [nt] 330 to 349; accession no. NM_012063, following advice from A. M. Van der Bliek, David Geffen School of Medicine, UCLA), 5′-GGATATTGAGCTTCAAATCA-3′ for D2 (nt 552 to 571; accession no. NM_012063). The specificity of each sequence was confirmed by BLAST searches. The oligonucleotides corresponding to these sequences were cloned into pSUPER-RETRO mammalian expression vector (kindly provided by Rewen Agami, The Netherlands Cancer Institute) as previously described [21]. To control for the potential side effects of transfecting cells with the pSUPER-RETRO vectors and expressing shRNAs, all Ctrl cells were transfected with firefly luciferase-targeted shRNA-expressing pSUPER-RETRO vector (sequence 5′-CGTACGCGGAATACTTCG A-3′) as described previously [44].

The production of vesicular stomatitis virus G, pseudotyped retroviral particles encapsulating the respective pSUPER-RETRO vectors was performed as previously described [18]. Briefly, 293T cells were transfected with the pSUPER-RETRO shRNA, pCMVgag/pol and pMD2G vectors. 48 h after transfection, the culture medium containing the viral particles was collected and centrifuged at 1,000×*g* for 10 min at 4°C, filtered through a 0.45 µm filter, and stored at −80°C. HeLa cells were transduced by incubating actively growing cultures with the viral supernatants for 16 to 24 h.

### Immunoblotting and immunocytochemistry

For immunoblotting, cells were resuspended in lysis buffer: 10 mM HEPES, 300 mM KCl, 5 mM MgCl_2_, 1 mM EGTA, 1% Triton X-100 (vol/vol), 0.1% (wt/vol) sodium dodecyl sulfate (SDS), pH 7.4, supplemented with 1× proteinase inhibitor mixture (Roche). Lysates were spun at 2,000×*g*, and the protein concentration was determined by Bradford assay (Bio-Rad). Equal amounts of protein were subjected to SDS-polyacrylamide gel electrophoresis, transferred to nitrocellulose membranes (Schleicher & Schuell), immunoblotted with primary antibodies followed by horseradish peroxidase-conjugated secondary antibodies, and developed via enhanced chemiluminescence.

Mitochondrial protein oxidation was determined in lysates of isolated mitochondria using the OxyBlot™ Protein Oxidation Detection Kit (Chemicon) according to the manufacturer's instruction. The anti-DNPH immunoblot was quantified and normalized to the loading control (VDAC) using ImageJ version 1.40 g.

Immunocytochemistry was performed as follows: cells grown on glass coverslips were fixed in 4% paraformaldehyde diluted in growth medium for 20 min at room temperature (RT) followed by PBS washes. The cells were then permeabilized with 0.1% Triton X-100 in PBS and blocked at RT in PBS containing 0.1% Triton X-100 and 5% normal goat serum. The coverslips were then incubated with primary antibodies diluted in blocking buffer for 2 h at RT (or overnight at 4°C) followed by washes in permeabilization buffer. Immunoreactive proteins were visualized by incubating the cells with fluorescein isothiocyanate (FITC) or Texas Red-coupled mouse or rabbit secondary antibodies (Vector Laboratories) in permeabilization buffer for 1 h at RT, followed by washes in permeabilization buffer. Coverslips were mounted in a DABCO solution (2.4% DABCO-52% glycerol in PBS; pH 7.2). Fluorescent images were visualized using a Zeiss Axiovert 135TV apparatus or an Olympus IX70 Deltavision Microscope. Images were captured using a charge-coupled-device camera and processed using Adobe Photoshop CS 8.0. Quantification of the integrated fluorescent intensity of individual mtDNA nucleoids was performed on maximal projection images of 9 D1 cells and 10 Ctrl cells using MetaMorph version 6.3r7.

Immunocytochemistry to quantify cell proliferation by BrdU incorporation was performed as previously described [26] on HeLa cells 120 hrs after transfection that had been incubated in medium containing 25 µM BrdU for 18 hrs. Briefly, coverslips were permeabilised as outlined above and DNA was denatured by incubating the coverslips in 2N HCl for 30 min at 37°C. The coverslips were washed extensively with 0.1 M Sodium Borate pH 8.5 and immunostained as above using anti-BrdU as the primary anitibody.

### ROS, ΔΨm, ATP and oxygen consumption measurements

The intracellular level of ROS and ΔΨm of shRNA transfected cells was measured by flowcytometry of live cells stained with 40 µM Carboxy-H_2_DCFDA or 0.5 µM carbocyanine dye 5,5′,6,6′,-tetrachloro-1,1,3,3′tetraethylbenzimidazolylcarbocyanine iodide (JC-1) or tetramethylrhodamine ethyl ester (TMRE). Briefly, adherent cells in culture (for H_2_DCFDA) or cells in suspension after trypsinisation (for JC1 and TMRE) were incubated with medium (or PBS containing 0.15 g/L CaCl_2_, 1 g/L glucose in the case of H_2_DCFDA) containing the respective dyes for 15 min (for TMRE) to 1 hr (for H_2_DCFDA and JC1). JC1 and TMRE stained cells were then analysed by flowcytometry in growth media and H_2_DCFDA stained cells were washed and collected by trypsinisation PBS containing 0.15 g/L CaCl_2_, 1 g/L glucose before FACS analysis. Of note in order to normalise the intensity of TMRE fluorescence for the volume of mitochondria, the latter was divided by the intensity of TMRE after addition of 100 µM for the mitochondrial uncoupler CCCP.

Cellular ATP levels were determined using the ATP determination kit (Molecular probes, Invitrogen) according to the manufacturer's instructions. Briefly, HeLa cells transfected for 96 hrs were collected by trypsinisation, counted and lysed (on ice of 10 min) at 10^5^ cells per 100 ul of PLB (Promega). The lysates were spun at 12,000×*g* at 4°C for 5 min to pellet cell debris. The protein concentration of the cleared lysates was determined as before and triplicates of 3 µg of lysates were used for ATP determination with a D-luciferin/firefly luceferase reaction mix and luminescence was measured on a CHAMELEON™ multi-plate reader and compared to a freshly prepared ATP standard curve.

Oxygen consumption measurements were made on live non-permeabilised retrovirally infected cells in growth medium (supplemented with 10 mM Hepes, pH 7.5) using a Gilson oxygraph equipped with a Clark electrode (Gilson Medical Electronic, Middleton, WI). Oxymetry on isolated organelles was performed on mitochondria isolated from retrovirally infected cells. Briefly, the cells were scraped in medium, washed once in cold PBS, and resuspended in MB (210 mM mannitol, 70 mM sucrose, 10 mM HEPES, pH 7.5, 1 mM EDTA) supplemented with protease inhibitors (Roche). Cells were broken by 15 strokes in a 2 ml glass homogeniser (Kontes Glass Co) on ice and the suspension was centrifuged at 500×*g* at 4°C for 5 min. The supernatant was kept, and the pellet was resuspended in homogenization buffer and homogenised as above. The suspension was spun as above and the supernatants were pooled and centrifuged for 5 min at 1,500×*g* at 4°C to pellet the remaining nuclei and unbroken cells. The supernatant was further centrifuged for 5 min at 10,000×*g* at 4°C to pellet the mitochondria. The supernatants were discarded, and the mitochondria were washed with homogenization buffer. The quantity of protein was determined as before and 100 µg of the mitochondrial fraction was resuspended in respiration buffer (210 mM Mannitol, 70 mM Sucrose, 1 mM EDTA, 4 mM Na_2_HPO4, 5 mM MgCl_2_) to determine the rate of oxygen consumption of the isolated organelles using the above Clark electrode system. State IV respiration, corresponding to the rate of uncoupled respiration, was assessed in the presence of 5 mM Succinate and the State III respiratory rate (corresponding to coupled respiration) was obtained after addition of 0.5 mM ADP to the 5 mM Succinate. The percentage of respiratory uncoupling was obtained by dividing oxygen consumption in the presence of 5 mM Succinate (State IV) by that after addition of 0.5 mM ADP (State III).

Flow cytometry was performed using a Becton Dickinson FACS Track flow cytometer with CellQuest software 3.3. FL1 and FL2 corresponds the 530/30 BP and 585/42 BP emission filters respectively.

### Quantification of mtDNA by Q-PCR

Quantification of the mtDNA was performed as previously described by Legros and colleagues [26]. Briefly, 100,000 cells were resuspended in 100 µl extraction solution (0.2 mg/ml proteinase K, 0.2% SDS and 5 mM EDTA in PBS) and incubated at 50°C for 3 h. Total DNA was then precipitated by addition of 10 µl of 3 M sodium acetate (pH 5.2), 110 µl isopropanol and incubation for 20 minutes on ice before centrifugation at 12,000 rpm at 4°C. The DNA-pellet was washed once with cold 70% ethanol, air dried for 15 min and resuspended in 100 µl TE buffer at 4°C overnight. Realtime PCR amplification was performed on 10 ng of total DNA using a iCycler (Bio Rad) and iQ SYBR Green Supermix (BioRad) following the manufacturer's instructions. A 211 bp fragment of the mtDNA 12S RNA gene was amplified between nucleotide 1095 and nucleotide 1305 (Forward primer: 5′ GCTCGCCAGAACACTACGAG 3′, reverse primer: 5′ CAGGGTTTGCTGAAGATGGCG 3′). Elongation translation factor 1 gene (EEF1A1) was used as an endogenous reference across all experimental conditions (Forward primer: 5′ GGATTGCCACACGGCTCACATT 3′, reverse primer: 5′ GGTGGATAGTCTGAGAAGCTCTC 3′).

## Supporting Information

Figure S1 Depleting HeLa cells of Drp1 using the D1 or D2 construct inhibits mitochondrial fission A. HeLa cells were transiently transfected with the Ctrl, D1 or D2 constructs, selected with puromycin for 24 h and collected for Western blotting analysis using the indicated antibodies 96 h after transfection. B. HeLa cells transfected with the Ctrl, D1 or D2 constructs and treated as in A. were immunostained with a rabbit TOM20 antibody 96 h after transfection. The scale bar corresponds to 15 µm.
(2.87 MB TIF)
