# Supplementary figures and images for: Preventing Mitochondrial Fission Impairs Mitochondrial Function and Leads to Loss of Mitochondrial DNA
Source: PLoS One. 2008 Sep 22;3(9):e3257. doi: 10.1371/journal.pone.0003257 (PMC2532749; doi:10.1371/journal.pone.0003257)

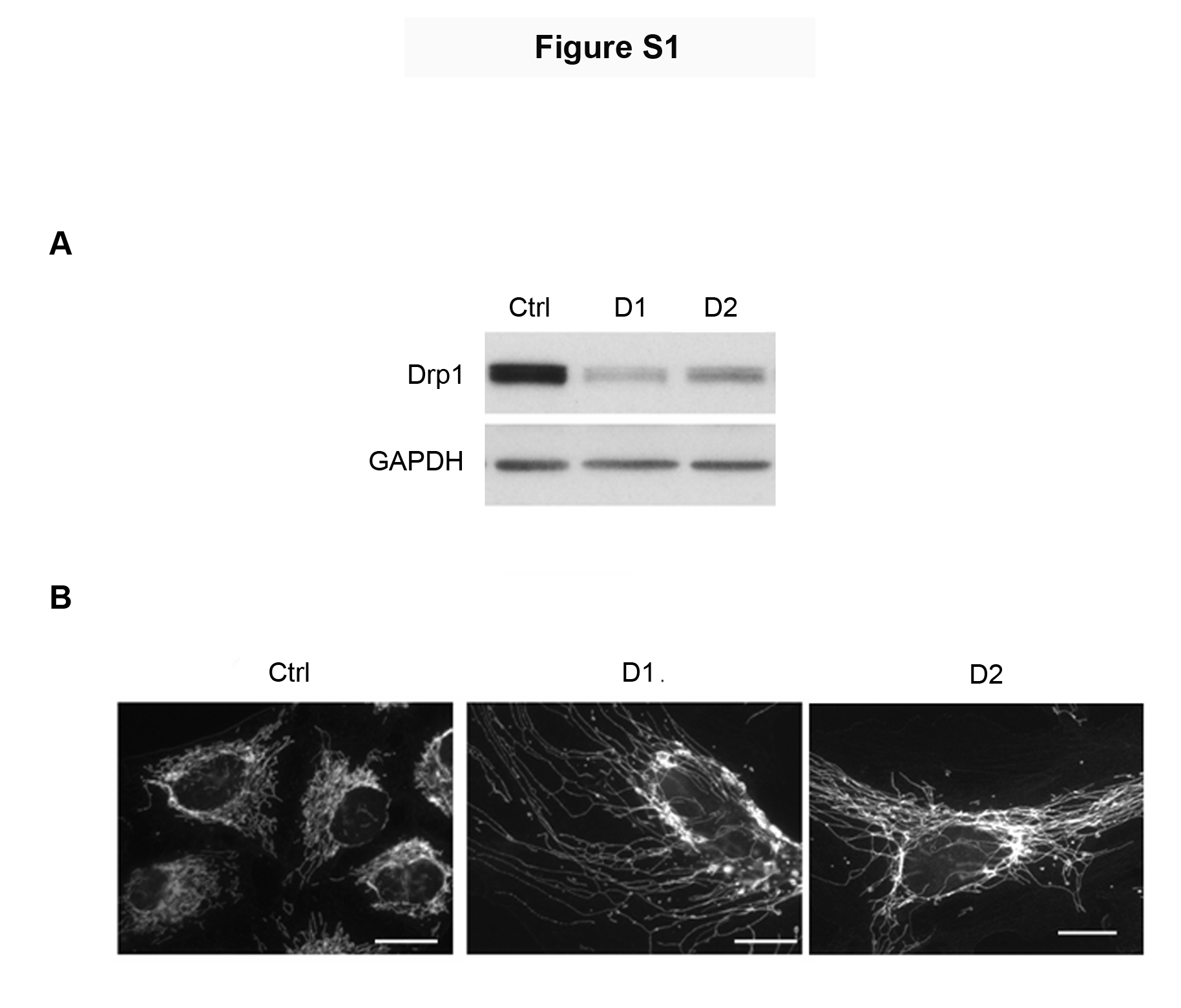

Supplement: Figure S1 — Depleting HeLa cells of Drp1 using the D1 or D2 construct inhibits mitochondrial fission A. HeLa cells were transiently transfected with the Ctrl, D1 or D2 constructs, selected with puromycin for 24 h and collected for Western blotting analysis using the indicated antibodies 96 h after transfection. B. HeLa cells transfected with the Ctrl, D1 or D2 constructs and treated as in A. were immunostained with a rabbit TOM20 antibody 96 h after transfection. The scale bar corresponds to 15 µm. (2.87 MB TIF) [file pone.0003257.s001.tif]
